# Clinical tumor stage is the most important predictor of pathological complete response rate after neoadjuvant chemotherapy in breast cancer patients

**DOI:** 10.1007/s10549-017-4155-2

**Published:** 2017-02-15

**Authors:** Briete Goorts, Thiemo J. A. van Nijnatten, Linda de Munck, Martine Moossdorff, Esther M. Heuts, Maaike de Boer, Marc B. I. Lobbes, Marjolein L. Smidt

**Affiliations:** 1 GROW - School for Oncology and Developmental Biology, Maastricht University Medical Centre, Maastricht, The Netherlands; 2 Department of Surgery, Maastricht University Medical Centre, P.O. Box 5800, 6202 AZ Maastricht, The Netherlands; 3 Department of Radiology and Nuclear Medicine, Maastricht University Medical Centre, Maastricht, The Netherlands; 4 Department of Research, Netherlands Comprehensive Cancer Organisation (IKNL), Utrecht, The Netherlands; 5 Department of Medical Oncology, Maastricht University Medical Centre, Utrecht, The Netherlands

**Keywords:** Breast cancer, Pathological complete response, Neoadjuvant chemotherapy, Tumor size, Survival

## Abstract

**Background:**

Pathological complete response (pCR) is the ultimate response in breast cancer patients treated with neoadjuvant chemotherapy (NCT). It might be a surrogate outcome for disease-free survival (DFS) and overall survival (OS). We studied the effect of clinical tumor stage (cT-stage) on tumor pCR and the effect of pCR per cT-stage on 5-year OS and DFS.

**Methods:**

Using the Netherlands Cancer Registry, all primary invasive breast cancer patients treated with NCT from 2005 until 2008 were identified. Univariable logistic regression analysis was performed to evaluate the effect of cT-stage on pCR, stepwise logistic regression analysis to correct for potential confounders and Kaplan–Meier survival analyses to calculate OS and DFS after five years.

**Results:**

In 2366 patients, overall pCR rate was 21%. For cT1, cT2, cT3, and cT4, pCR rates were 31, 22, 18, and 17%, respectively. Lower cT-stage (cT1-2 vs cT3-4) was a significant independent predictor of higher pCR rate (*p* < 0.001, OR 3.15). Furthermore, positive HER2 status (*p* < 0.001, OR 2.30), negative estrogen receptor status (*p* = 0.062, OR 1.69), and negative progesterone receptor status (*p* = 0.008, OR 2.27) were independent predictors of pCR. OS and DFS were up to 20% higher in patients with cT2-4 tumors with pCR versus patients without pCR. DFS was also higher for cT1 tumors with pCR.

**Conclusions:**

The most important predictor of pCR in breast cancer patients is cT-stage: lower cT-stages have significantly higher pCR rates than higher cT-stages. Patients with cT2-4 tumors achieving pCR have higher OS and DFS compared to patients not achieving pCR.

## Introduction

There is an increase of neoadjuvant chemotherapy (NCT) administration in breast cancer patients, compared to adjuvant chemotherapy [1]. The most important aims of this strategy are to improve surgical safety and to minimize the extent of the operation by downsizing the tumor. As a result, breast-conserving surgery rates have increased since the introduction of NCT [1].

Pathological complete response (pCR), i.e., absence of any residual cancer, is the ultimate response to NCT in breast cancer patients. Unfortunately, only 22% of all patients treated with NCT achieves pCR of the breast tumor [2]. Patients that achieve pCR show improved survival rates as compared to patients without pCR for all subtypes except for low-grade estrogen receptor (ER)-positive and human epidermal growth factor receptor 2 (HER2)-negative subtypes [3–11]. Therefore, it is currently being debated whether pCR can be considered as a surrogate outcome for disease-free survival (DFS) and overall survival (OS) [3].

Ideally, patients achieving pCR should be identified before NCT administration. This way, determining the ultimate patient-tailored treatment plan would be possible at initiation of treatment. For example, the possibility to perform breast conserving surgery after NCT would be clear from the start. Currently, we are not able to identify those patients. Literature shows that high tumor grade, positive HER2 status, negative ER status, and negative progesterone receptor (PR) status increase the probability of achieving pCR [2–6, 12, 13]. With these beneficial factors to achieve pCR in mind, clinicians can make a rough estimation of the chance of an individual patient achieving pCR. However, we need to search for additional factors that contribute to the fine tuning of this estimation.

One other factor that might contribute to the probability of achieving pCR is tumor size. Earlier research of 144 patients treated with NCT between 1975 and 1996 shows that smaller tumors are more likely to respond to NCT [14]. A study of Bonadonna and colleagues shows that the degree of response is inversely proportional to the initial tumor size in tumors larger than three centimeters [15]. The estimation of pCR chances might therefore be more accurate taking tumor size into account.

The aim of this study was to investigate the effect of tumor size, expressed as clinical tumor stage (cT-stage), on pCR with correction for potential confounders like high tumor grade, positive HER2 status, negative ER status, and negative PR status. Since it is currently being debated whether pCR can be considered as a surrogate outcome for disease-free survival (DFS) and overall survival (OS) in a selected group of patients, the effect of pCR per cT-stage on DFS and OS was analyzed as well.

## Methods

### Study design and patient selection

Using data from the Netherlands Cancer Registry (NCR), a nationwide, population-based cancer registry managed by the Netherlands Comprehensive Cancer Organisation (IKNL), all patients with primary invasive epithelial breast cancer treated with NCT in the Netherlands between January 2005 and December 2008 were identified and considered for inclusion. Exclusion criteria were synchronous breast cancer or distant metastases at time of diagnosis, previous invasive breast cancer, neoadjuvant radiotherapy, and neoadjuvant endocrine therapy.

### Data collection

The NCR data were retrieved from patients’ records by trained data registrars. They collected data on patient and tumor characteristics (age, grade, tumor type, clinical and pathological TNM-stage, ER, PR, and HER2 status), and on surgical, radiation and systemic treatment of all new breast cancer patients directly from the medical records in all Dutch hospitals. An active follow-up was conducted registering the first recurrence (local, regional or distant metastasis) within 5 years after diagnosis. Additional data on date of death and date of emigration were derived from the Municipal Personal Records Database, complete until 31 December 2014.

### Neoadjuvant systemic treatment, surgical procedure, and pathological analysis

In addition to neoadjuvant chemotherapy, neoadjuvant targeted therapy could be administered in case of HER2 receptor amplification (trastuzumab). Furthermore, all patients underwent surgery of the breast and ipsilateral axilla. Breast surgery consisted of breast conserving therapy or mastectomy, depending on clinical tumor size, breast size, and patient preference. Axillary surgery consisted of sentinel lymph node biopsy in case of cN0 and axillary lymph node dissection in case of cN+. Pathological analyses of pre-treatment core biopsies and surgically removed tissue were performed locally in accordance to the Dutch breast cancer guideline at the time of diagnosis [16]. Patients were classified as positive for ER or PR when ≥10% of the tumor cells showed positive nuclear staining [16, 17].

### Objectives and endpoints

Our primary endpoint was complete remission or pCR, defined as absence of macroscopic and microscopic evidence of invasive tumor in the resected breast tissue (ypT0 or ypTis). In this study, pathological response of the breast tumor to NCT was studied, not the response of the axillary lymph nodes. To evaluate the effect of tumor size on pCR, the parameter cT-stage was used because the exact tumor diameter on preoperative imaging was not registered.

Secondary endpoints were DFS and OS. DFS is a composite endpoint consisting of locoregional recurrence, distant metastases (defined according to the consensus-based event definitions for recurrence classification of Moossdorff et al.) [18], contralateral breast cancer, or death within five years. Survival time was defined as time between date of diagnosis and any of the above-mentioned endpoints whichever occurred first. In OS, survival time was defined as time between date of diagnosis and death. For DFS, events occurring within 3 months of date of diagnosis were considered to be synchronous to the primary tumor and were not considered to be events.

### Statistical analyses

The number of patients achieving pCR per cT-stage and breast cancer subtype was studied. Univariable logistic regression was performed to evaluate the effect of lower cT-stage on pCR and to evaluate the effect of possible confounders high grade (grade 3 vs grade 1–2), positive HER2 status, negative ER status, and negative PR status on pCR. Since not all patients with HER2 amplification received targeted therapy between 2005 and 2008, a separate analysis was performed for HER2-positive patients who received targeted therapy versus those who did not. Stepwise logistic regression was executed to correct for possible confounders. Kaplan–Meier survival analyses were used to calculate OS and DFS. A *p* value of <0.05 was considered statistically significant. Statistical analyses were performed using Statistical Package for the Social Sciences (SPSS), version 22.0 (IBM Corporation, Armonk, NY, USA).

## Results

A total of 2366 primary invasive epithelial breast cancer patients treated with NCT were included. Baseline characteristics are shown in Table 1.

**Table 1:** Baseline characteristics

	*N* (%)
Total number of patients	2366 (100.0)
Median age in years [range]	49 [21–86]
ER	
Positive	1483 (62.7)
Negative	842 (35.6)
Unknown	41 (1.7)
PR	
Positive	1090 (46.1)
Negative	1163 (49.1)
Unknown	113 (4.8)
HER2	
Positive	669 (28.3)
Negative	1561 (66.0)
Unknown	136 (5.7)
Grade	
1	99 (4.2)
2	326 (13.8)
3	507 (21.4)
Unknown	1434 (60.6)
Operation	
Lumpectomy	531 (22.4)
Mastectomy	1835 (77.6)
Adjuvant therapy	
Radiotherapy	2003 (84.7)
Hormonal therapy	1393 (58.9)

*ER* estrogen receptor, *PR* progesterone receptor, *HER2* human epidermal growth factor receptor 2

In 320 patients, clinical and/or pathological tumor stages were unknown. In 420 of the remaining 2046 patients, histopathology showed pCR (20.5%). In cT1 patients, 58 of 187 (31.0%) reached pCR, so did 186 of 829 (22.4%) cT2 patients, 94 of 534 (17.6%) cT3 patients, and 82 of 496 (16.5%) cT4 patients. The distribution of breast cancer subtypes differs slightly per cT-stage, the percentage of the hormone receptor positive subtypes decreases, and the percentage of the hormone receptor negative subtypes increases with higher cT-stage, especially the ER−/PR−/HER2+ subtype increases (Table 2).

**Table 2:** Pathological tumor response of patients receiving neoadjuvant chemotherapy per clinical tumor stage and per breast cancer subtype

cT-stage	*N* (% of total N per cT-stage)	ypT0 or pCR (%)	ypT1 (%)	ypT2 (%)	ypT3 (%)	ypT4 (%)
cT1	187	58 (31.0)	108 (57.8)	17 (9.1)	3 (1.6)	1 (0.5)
ER/PR+HER2+	30 (16.0)	14 (46.7)				
ER/PR+HER2−	93 (49.7)	12 (12.9)				
ER, PR−HER2+	12 (6.4)	6 (50.0)				
Triple negative	34 (18.2)	20 (58.8)				
Unknown	18 (9.6)	6 (33.3)				
cT2	829	186 (22.4)	324 (39.1)	289 (34.9)	24 (2.9)	6 (0.7)
ER/PR+HER2+	123 (14.8)	42 (34.1)				
ER/PR+HER2−	419 (50.5)	37 (8.8)				
ER−PR−HER2+	77 (9.3)	35 (45.5)				
Triple negative	151 (18.2)	60 (39.7)				
Unknown	59 (7.1)	12 (20.3)				
cT3	534	94 (17.6)	141 (26.4)	176 (33.0)	119 (22.3)	4 (0.7)
ER/PR+HER2+	66 (12.4)	20 (30.3)				
ER/PR+HER2−	260 (48.7)	16 (6.2)				
ER−PR−HER2+	71 (13.3)	25 (35.2)				
Triple-negative	100 (18.7)	26 (26.0)				
Unknown	37 (7.0)	7 (18.9)				
cT4	496	82 (16.5)	112 (22.6)	126 (25.4)	71 (14.3)	105 (21.2)
ER/PR+HER2+	62 (12.5)	11 (17.7)				
ER/PR+HER2−	210 (42.3)	6 (2.9)				
ER−PR−HER2+	95 (19.2)	39 (41.1)				
Triple-negative	100 (20.2)	21 (21.0)				
Unknown	29 (5.9)	5 (17.2)				

*cT-stage* clinical tumor stage, *pCR* pathologic complete response, *ypT0–ypT4* pathological tumor stage after chemotherapy 0 to 4, *ER* estrogen receptor, *PR* progesterone receptor, *HER2* human epidermal growth factor receptor 2

In univariable regression analyses, lower cT-stage (cT1-2 vs cT3-4) was a significant predictor of higher pCR rate (*p* < 0.001). High grade (grade 3 vs grade 1–2) (*p* = 0.001), positive HER2 status (*p* < 0.001), negative ER status (*p* < 0.001), and PR receptor status (*p* < 0.001) were significant predictors of higher pCR rates as well (Table 3). Multivariable analysis demonstrated lower cT-stage, positive HER2 status, and negative PR receptor status as being independent predictors of pCR. Lower cT-stage was the most important predictor with an odds ratio of 3.154 (*p* < 0.001) (Table 3).

**Table 3:** Univariable and multivariable analysis of predictors of pathologic complete response with their pathologic complete response rates

	%pCR	Univariable *p* value	Multivariable *p* value	Multivariable OR	95% CI for OR	
					Lower	Upper
cT-stage						
1–2	24.0	<0.001	<0.001	3.154	2.027	4.907
3–4	17.1					
Grade						
3	20.0	0.001	0.177	1.383	0.864	2.214
1–2	11.4					
ER						
Neg	36.3	<0.001	0.062	1.687	0.974	2.920
Pos	12.0					
PR						
Neg	29.3	<0.001	0.008	2.269	1.243	4.141
Pos	12.0					
HER2						
Pos	35.6	<0.001	<0.001	2.299	1.493	3.540
Neg	14.9					

*pCR* pathologic complete response, *OR* odds ratio, *cT-stage* clinical tumor stage, *ER* estrogen receptor, *PR* progesterone receptor, *HER2* human epidermal growth factor receptor 2

In 115 out of 669, HER2 positive patients pathological tumor stage was unknown. Separate analysis of 554 patients with a positive HER2 status showed an overall pCR rate of 35.6%. In patients receiving NCT including targeted therapy, this was 47.4% (*n* = 293) versus 22.2% in patients not receiving neoadjuvant targeted therapy added to chemotherapy (*n* = 261; *p* < 0.001). The pCR rate of patients with a positive HER2 status not receiving neoadjuvant targeted therapy was higher than pCR rate of patients with a negative HER2 status (14.9%). The pCR rate decreased with increasing cT-stage in both subgroups (Table 4).

**Table 4:** Number of HER2-positive breast cancer patients treated with versus without neoadjuvant targeted therapy that has pathologic complete response per tumor stage

cT-stage	N pCR/total with TT (%)	N pCR/total without TT (%)
cT1	11/20 (55.0)	9/22 (40.9)
ER/PR+	8/14	6/16
ER−, PR−	3/6	3/6
cT2	58/117 (49.6)	19/87 (21.8)
ER/PR+	30/67	12/56
ER−, PR−	28/49	7/28
cT3	29/66 (43.9)	18/74 (24.3)
ER/PR+	12/30	8/36
ER−, PR−	16/34	9/37
cT4	40/87 (46.0)	10/73 (13.7)
ER/PR+	8/26	3/36
ER−, PR−	32/58	7/37
cTx	1/3 (33.3)	2/5 (40.0)
Total	139/293 (47.4)	58/261 (22.2)

*cT-stage* clinical tumor stage, *pCR* pathological complete response, *TT* targeted therapy, *ER* estrogen receptor, *PR* progesterone receptor

**Fig. 1:**
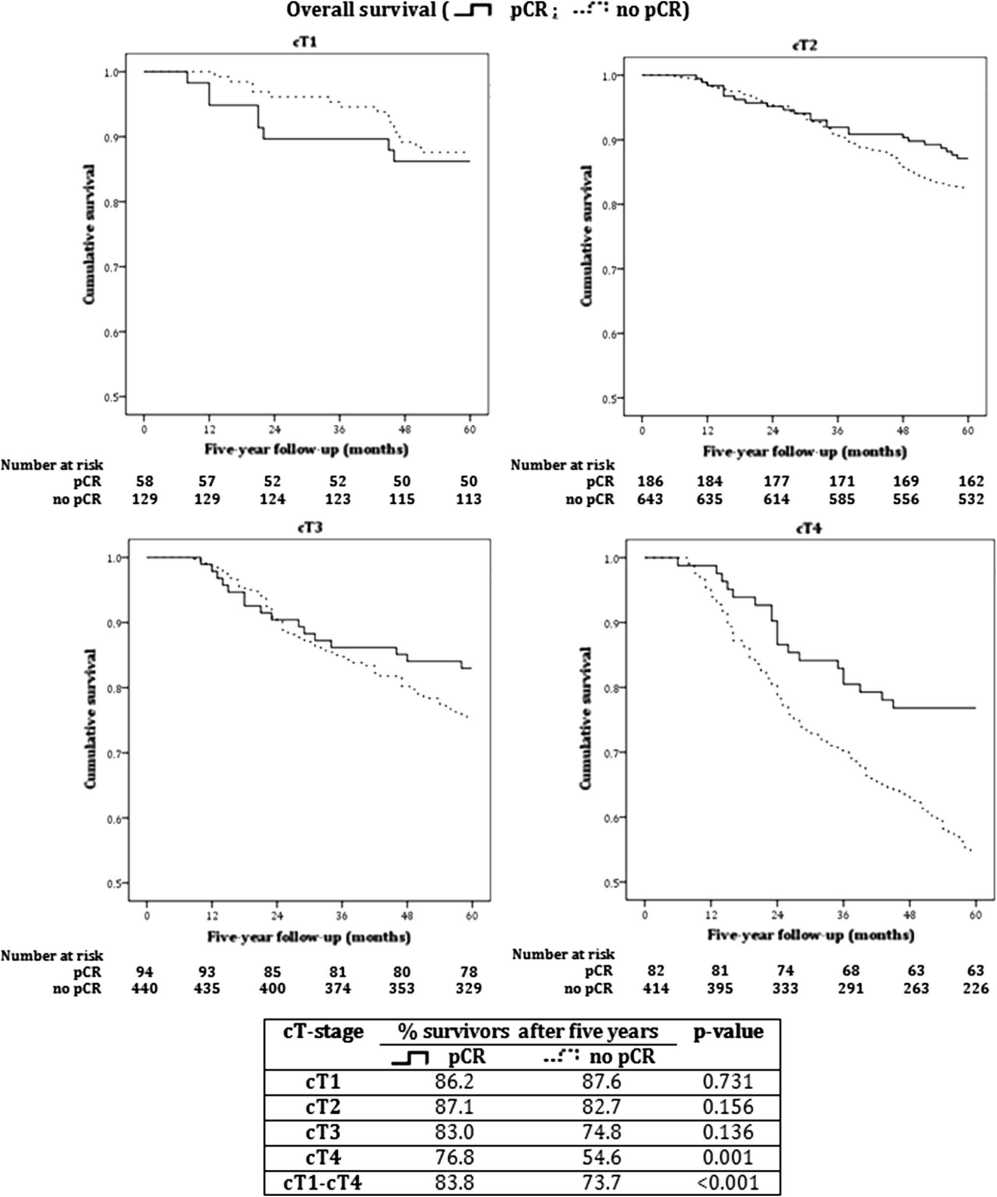
Kaplan–Meier curves 5-year overall survival of patients with versus without pathologic complete response per clinical tumor stage. *cT(-stage)* clinical tumor stage, *pCR* pathological complete response

Kaplan–Meier survival analyses demonstrated a 5-year OS of 76.5% for all patients receiving NCT, being 88.2% for cT1, 84.3% for cT2, 77.0% for cT3, and 58.8% for cT4 tumors. Furthermore, in patients with pCR 5-year OS was 83.8% versus 73.7% without pCR (*p* < 0.001). Also, all separate cT-stages except for cT1 showed a positive effect of pCR on OS but this difference was only statistically significant in cT4 (Fig. 1).

**Fig. 2:**
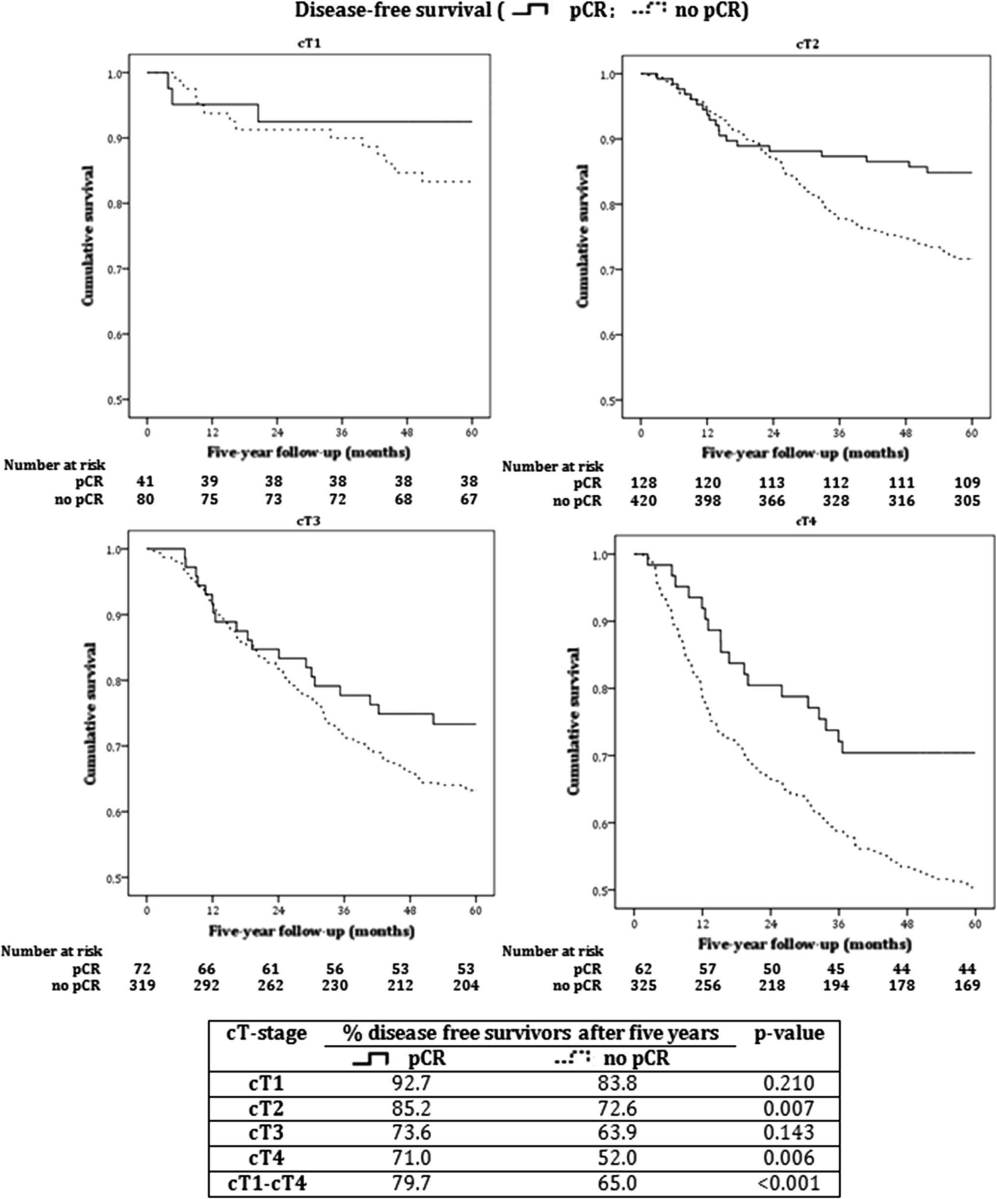
Kaplan–Meier curves of five year disease-free survival of patients with versus without pathologic complete response per clinical tumor stage. *cT(-stage)* clinical tumor stage, *pCR* pathological complete response

Additionally, Kaplan–Meier survival analyses showed that 5-year DFS was 68.6% for the entire group and 87.3% for cT1, 75.0% for cT2, 66.6% for cT3, and 55.9% for cT4 tumors. In patients with pCR, 5-year DFS was 79.7%, without pCR this was 65.0% (*p* < 0.001). Furthermore, the 5-year DFS was 9–19% higher in the pCR group versus the non-pCR group per cT-stage, but this difference was only statistically significant in cT2 and cT4 (Fig. 2).

## Discussion

The primary aim of this study was to investigate the effect of cT-stage on pCR to analyze if tumor size helps clinicians estimate pCR chances. Our results demonstrate that lower cT-stages have significantly higher pCR rates than higher cT-stages (cT1-2 vs cT3-4; *p* < 0.001). In case of cT1, cT2, cT3, and cT4, pCR rates were 31, 22, 18, and 17%, respectively. The cT-stage is an independent and stronger predictor of pCR than ER, PR, and HER2 status and grade. PCR decreases with increasing cT-stage, even though the percentage of hormone receptor-negative subtypes (which respond better to NCT than positive subtypes) increases with increasing cT-stage. The secondary aim was to analyze the effect of pCR per cT-stage on DFS and OS. Our results show that patients with cT2-4 breast tumors and pCR had an up to 20% higher (disease-free) survival rate compared to patients without pCR.

To the best of our knowledge, the relation between breast tumor size and pCR has never been studied before. An earlier study of Gajdos et al. (*n* = 138) did demonstrate that smaller tumors are more likely to respond to chemotherapy than larger tumors [14], and the study of Bonadonna et al. (*n* = 165) showed that the degree of response is inversely proportional to initial tumor size for tumors larger than 3 cm [15]. Furthermore, a study by Caudle and colleagues showed that large tumor size is a pre-treatment predictor of disease progression [19]. Jin et al. found that HER2 negative breast cancer patients with smaller tumor sizes were more likely to achieve pCR than the ones with larger tumor sizes [20].

The results of our study, which are very much along the lines of the above described earlier studies, emphasize the importance for clinicians to take cT-stage into account when estimating the chance of pCR in an individual patient. This study shows for example that a patient with a triple-negative tumor would have a chance of pCR of approximately 40% with a cT2 tumor and 26% with a cT3 tumor.

Furthermore, this study encourages clinicians to use NCT in early-stage breast cancer patients. The current Dutch guidelines recommend NCT in patients with a tumor larger than 2 cm or smaller high-risk tumors with as main goal a safer and less extensive surgery [16]. But as the oncologic field moves toward minimally invasive surgery or even watchful waiting [21, 22], breast cancer patients especially with small tumors (cT1-2) and higher chances of attaining pCR might be the perfect candidates for safe watchful waiting in the future. Future research goals are therefore to explore the possibilities for safe watchful waiting in breast cancer patients that achieve pCR and therewith to search for an imaging technique that is good enough to estimate the precise residual tumor size and predict pCR [23].

Multiple (retrospective) studies demonstrated that pCR can be used as surrogate outcome for OS and DFS [4–8] The recent meta-analysis of Cortazar analyzed the effect of pCR between treatment groups on event-free survival (EFS) and OS [3]. They found an association between pCR and improved EFS and OS but an increase in frequency of pCR between treatment groups could not be validated as a surrogate endpoint for improved EFS and OS. This may be explained by the heterogeneity of the treatment regimens in the studies in this meta-analysis, obscuring the association between pCR and survival.

In our study, patients with pCR of the breast tumor had an up to 20% higher (disease-free) survival rate compared to patients without pCR. This positive effect of pCR on 5-year OS was not present for cT1 tumors and only statistically significant in cT2 (DFS) and cT4 (DFS and OS) tumors. One potential explanation is that the prognosis of most cT1 breast tumors is already so favorable that their benefit of achieving pCR is only small. Another potential explanation which especially accounts for cT1 tumors is that selection for NCT probably differs from cT2-4 tumors, and it concerns only a small group of patients. In cT1 tumors, the choice to start NCT will more often be based on the presence of tumor positive lymph nodes, HER2 amplification or their triple-negative character; in the other groups, this choice will be more often based on tumor size, making it prognostically different subgroups. Patients with tumor-positive lymph nodes, amplification of HER2, or a triple-negative tumor have a worse prognosis than patients without these traits. Furthermore, looking at our Kaplan–Meier curves, we expect survival differences between patients achieving versus not achieving pCR only to increase by the years and 10-year survival differences to be statistically significant for all cT-stages. This should be investigated in future research. Nevertheless, achieving pCR will not entirely eliminate recurrence.

Our study was limited by its retrospective design. Chemotherapy regimens can affect pCR rate [5, 24]. Unfortunately, the exact regimens (number of treatments, dosages) were not registered in the NCR. Furthermore, because tumor grade often was unknown, this may have influenced the prognostic value of tumor grade or other factors in the multivariable analysis. Finally, in situ components in the resected tissue were not coded. Therefore, pCR was defined as no microscopic evidence of invasive tumor in our study. According to the literature, there might be a small difference in prognosis between patients with and without in situ components after NCT but this could not be studied [7].

In conclusion, the most important predictor of pCR is the cT-stage: lower cT-stages have significantly higher pCR rates than higher cT-stages, independent of grade, ER, PR, and HER2 status. Furthermore, 5-year OS and DFS were up to 20% higher in patients with cT2–cT4 tumors with pCR versus patients without pCR.
